# Towards transient space-use dynamics: re-envisioning models of utilization distribution and their applications

**DOI:** 10.1186/s40462-025-00538-5

**Published:** 2025-02-28

**Authors:** Yun Tao, Valeria Giunta, Luca Börger, Mark Q. Wilber

**Affiliations:** 1 Institute of Bioinformatics, University of Georgia, Athens, GA 30602 USA; 2 School of Mathematics and Computer Science, Swansea University, Swansea, SA1 8EN UK; 3 Department of Biosciences, Swansea University, Swansea, SA1 8EN UK; 4 Centre for Biomathematics, Swansea University, Swansea, SA1 8EN UK; 5 School of Natural Resources, University of Tennessee Institute of Agriculture, Knoxville, TN 37998 USA

**Keywords:** Utilization distribution, Transient dynamics, Space-use pattern, Home range, Territory, Disease ecology, Epidemiology

## Abstract

**Background:**

Models of utilization distribution in the form of partial differential equations have long contributed to our understanding of organismal space use patterns. In studies of infectious diseases, they are also being increasingly adopted in support of epidemic forecasting and scenario planning. However, as movement research shifts its focus towards large data collection and statistical modeling of movement trajectories, the development of such models has notably slowed.

**Methods:**

Here, we demonstrate the continued importance of modeling utilization distribution to predict variation in space-use patterns over time. We highlight the considerable, yet largely untapped, potential of such models, which have historically been limited by the steady-state assumption due to longstanding technical constraints. Now, by adapting existing computational tools primarily developed for material science and engineering, we can probe beyond the steady states and unlock from them a broad spectrum of complex, transient space-use dynamics. Our approach requires little experience in numerical analysis and is readily accessible to model practitioners in ecology and epidemiology across diverse systems where movement is a critical feature.

**Results:**

We illustrated our approach using a mix of canonical and novel case studies, covering topics from wildlife translocation to vaccine deployment. First, we revisited a classical model of canid territorial formation driven by scent-mediated conspecific avoidance. Transient space-use analysis uncovered previously hidden spatial dynamics that are ecologically informative. Next, we applied our approach to long-distance movement on realistic landscapes. Habitat and land-use heterogeneities markedly affected the transient space-use dynamics and short-term forecasts, even when the steady state remained unchanged, with direct implications for conservation management. Finally, we modeled transient space-use dynamics as both a response to and a driver of transient population dynamics. The importance of this interdependence was shown in the context of epidemiology, in a scenario where the movement of healthcare personnel is influenced by local outbreak conditions that are stochastically evolving.

**Conclusions:**

By facilitating transient space-use analysis, our approach could lead to reevaluations of foundational ecological concepts such as home range and territory, replacing static with dynamic definitions that more accurately reflect biological realities. Furthermore, we contend that a growing interest in transient space-use dynamics, spurred by this work, could have transformative effects, stimulating new research avenues in ecology and epidemiology.

**Supplementary Information:**

The online version contains supplementary material available at 10.1186/s40462-025-00538-5.

## Background

Historically, quantitative studies of animal movement in the context of ecology have frequently focused on capturing animal space-use patterns, i.e., the geographic region where individuals or groups are likely found. Among the common measures of space-use pattern (e.g., minimum convex polygon, local convex hull), utilization distribution (UD) holds particular importance in the development of animal movement theory. A UD explicitly describes an individual’s probability of being at a location at any given time [1]. It is conventionally used to quantify area-restricted space-use patterns, i.e., a home range or a territory, that one or multiple animals presumably form and maintain during the study period.

Models of UDs have been extensively developed to understand the relationship between specific movement mechanisms and the spatial structure of UDs [1–3]. Most early models follow basic principles of statistical mechanics and are formulated as Fokker–Planck equations, a class of partial differential equation that describes how a probability density function, e.g., a UD, evolves over time. In the past three decades, they have deepened our insights into the causes, processes, and ecological consequences of animal movement and gave mechanistic explanations to widely observed phenomena such as territorial separation [4, 5], predator–prey distribution [6], and home range expansion/contraction [7]. In an era when acquiring accurate movement data was both prohibitively expensive and technically challenging, these mechanistic (“classical”) space-use models played a critical role in laying the theoretical groundwork that gave rise to the modern field of movement ecology.

At the start of the millennium, major advances in bio-logging and tracking technology facilitated a surge in high-resolution movement data [8, 9]. As the collection of these data became more accurate and cost-effective, the emphasis in movement modeling shifted from predicting space-use patterns to estimating actual movement trajectories (or paths). Concurrently, the development of statistical movement models accelerated, filling missing details in movement trajectories often by integrating additional data streams, including biometrics, meteorological data, and habitat indices [10–12]. New frameworks for statistical modeling emerged, including state-space models [13, 14], resource-selection analysis [15], and integrated step-selection analysis [16], all of which are adept at inferring key determinants of movement decisions and predicting changes in movement trajectories (see [17] and references therein). The application of Fokker–Planck equations shifted as well, simple expressions, for instance, ones that exclude non-diffusive movement, are now routinely used as process models in a hierarchical Bayesian setting, fitted to observational data to predict population and disease spread [18–20]. In contrast, classical space-use models and, by extension, the interest in forecasting UDs, appear to have receded from the mainstream of movement research (but see [21, 22]).

Nevertheless, UD as a concept remains essential for gaining a holistic view of an animal’s spatial behavior [3, 23, 24]. Rather than pinpointing the exact locations of a given individual over time, UD provides an comprehensive measure that can encompass the entire space an individual might occupy over a timescale much longer than the observation period. UD may also describe a space occupied by multiple individuals, both observed and unobserved, sharing similar movement behaviors. In essence, it represents the aggregate outcome of many realized and potential movement trajectories. Because a UD inherently accounts for a large number of possibilities, the resulting inferences are less susceptible to the confounding effects of process randomness and measurement errors that commonly affect trajectory estimation. UDs are therefore well-suited for drawing ecological generalities, which may inform conservation strategies for managing hypothetical, future scenarios not yet empirically studied.

Beyond ecological applications, UD has become a useful tool in predicting and managing the spread of infectious diseases [25–29], functioning similarly to the concept of activity space [30]. Unlike in wildlife ecology in which the interest primarily lies in estimating where an animal is most likely present, disease ecology and epidemiology are generally motivated by risk assessment, an objective that requires knowing not only areas where hosts tend to visit, but also less frequented areas where transmission potential is elevated due to spatial and demographic factors (e.g., contact structure, geographic variations in pathogen viability, local seroprevalence, localized behaviors). By encompassing areas that hosts rarely visit, UD predictions enable us to identify high-risk areas that are difficult to detect from population monitoring alone. Moreover, policy decisions in response to a novel outbreak often need to be made amidst information uncertainty, including a lack of movement data that could have implications for management decisions and success. Indeed, current studies suggest that modeling UDs based on simple, general movement assumptions could help optimize surveillance systems in spillover prevention [31] and inform deployment strategies in vaccine rollout [32]. As more movement and mobility data are incorporated into disease forecasts, the use of UD in disease modeling will likely grow in the coming years.

In recent years, much of the progress in our understanding of UD has been driven by statistical ecology. While traditional UD estimation methods, notably kernel density estimator (KDE) and convex hull-based approaches (e.g., minimum convex polygon), do not explicitly account for temporal information in movement data, vastly improved versions are now widely available. For example, autocorrelated KDE (AKDE) addresses an individual’s positional autocorrelation and involves methods that could characterize non-stationary space use [33, 34], temporal adaptive KDE (TAKDE), on the other hand, applies a sliding window mechanism to achieve real-time UD estimation [35]. Comparatively, PDE-based models, a “principal workhorse” that can predict variations in both space and time in a unified manner [22], have been under-exploited. Yet, they offer several distinct benefits: the equations provide clearly defined movement mechanisms in relation to biotic and abiotic factors; the models facilitate the detection of emergent properties of complex ecological systems; and perhaps more importantly, the framework is fundamentally predictive, hence, it can support the forecasts of species responses to global change. In this study, we adopt a PDE-based approach and highlight the need for a renewed focus on this classical modeling framework.

In classical space-use models, UDs are generally characterized solely by the steady-state (equilibrium) solutions, depicting the stabilized space-use pattern as time approaches infinity. Transient (nonstationary) UD dynamics, i.e., how the pattern evolves over time *before* stabilization, were seldom explored. This gap stems partly from the significant technical challenges involved in obtaining the transient solutions of Fokker–Planck equations, a nontrivial task that required substantial expertise in numerical analysis (refer to Box for prior efforts to circumvent these challenges). Nonetheless, increasing evidence suggests that space-use patterns exist mostly in a state of transition [7, 36, 37]. By predominantly focusing on the steady states, previous models may have overlooked significant amounts of transitional details. The problem becomes more pressing in light of recent studies suggesting that forecasts of transient, sometimes short-lived, space-use patterns can better inform conservation and outbreak management actions than static, long-term forecasts [38, 39]. For example, when controlling newly invading pest animals, predicting their transient space-use dynamics, e.g., slow diffusion immediately after introduction followed by rapid dispersal [40], can reveal a brief grace period in which spatially targeted interventions are highly effective.

As is evident from numerous publications (e.g., [39, 41–43]), the importance of modeling transient dynamics is widely acknowledged in population, community, and disease ecology. Here, we extend the discussion to movement ecology with the goal of cultivating new perspectives and promoting real-time forecasts of movement-driven systems [39, 44]. In particular, we demonstrate that, without transient analysis, the full potential of UD remains largely untapped.

In this paper, we modeled transient UDs mechanistically in the classical framework, free from the conventional constraints of steady state. The models can capture an individual’s probable locations at any timescale, including its long-term area coverage. By giving a consistent interpretation of UD independent of time, we effectively bridged two opposing concepts in statistical ecology: occupancy distribution and range distribution [33]. Our approach is computational, mathematically tractable, and broadly accessible. Spatially explicit, complex interactions, e.g., stigmergy [45], can be readily represented in the model equations, which are solvable over arbitrary duration and spatial domain. Using only basic movement information, we may generate temporally detailed space-use forecasts. This low input requirement enables powerful inferences even when empirical movement data is sparse or of inconsistent quality, situations where statistical movement models are greatly disadvantaged.

Re-envisioning models of UDs from the perspective of transient dynamics motivate new lines of research. For instance, by charting the system's behavior over time, transient solutions permit investigation into the relative timing of movement-related phenomena. To demonstrate, we revisit a classical space-use model built on the assumption of scent-mediated conspecific avoidance [5, 46, 47], questioning whether scent marks will continue to accumulate after territories have been established. Additionally, hidden dynamics in classical space-use models can now be unlocked in search of general patterns. We illustrate this by uncovering new dynamical features from the previous example, modeled at a higher spatial dimension and with more individuals.

Transient space-use analysis can also support environmental impact assessment, particularly in conservation. Environmental disturbances such as extreme weather events or local resource depression often exert negligible long-term impacts on an animal’s space-use pattern or bear little relevance to immediate management decisions. Instead, impacts over shorter, more ecologically relevant timescales may be far more pronounced and even meaningful [48, 49]. We demonstrate this scenario by modeling an individual as it travels, while foraging, across a heterogeneous landscape, and then assessing the differences in transient space-use patterns with and without land use changes.

Transient space-use dynamics can simultaneously be a response to and a driver of transient population dynamics in space [50, 51]. Modeling this cross-scale interaction between the movement of individual units and population densities has diverse applications. As an example, to control an epidemic, decision-makers may need to adapt the deployment strategies for healthcare workers to take into account local outbreak intensities (densities of new infections). What movement rules to follow once the workers are in the field, e.g., prioritizing the protection of regions most at risk, might critically influence overall response effectiveness [52]. We explore this sample question by modeling the deployment of a vaccination team into a human population experiencing an outbreak, using a classical space-use model in conjunction with stochastic, agent-based simulations.

Our approach to finding transient solutions employs FiPy, an actively maintained, open-source PDE solver that can be applied to a wide range of Fokker–Planck equations. Alternative PDE solvers and their attributes are listed in Supplementary Information for further reference. Our case studies offer detailed examples of transient space-use analysis at a level of abstraction appropriate for most ecologists and epidemiologists with respect to numerical expertise. To demonstrate the degree of spatial complexity and insights achievable even with simple movement mechanisms, we limit our case studies to systems where all individuals display a centralizing tendency and directional preferences toward single fixed-point attractors.

Box: Competing approaches to modeling transient space-use dynamics Agent-based models are another common approach for modeling individual-level movement processes (e.g., [71]). They simulate the movements of one or more individuals on a finite landscape, the step length and direction of each movement decision can be determined by ecological interactions as well as by spatial memory (see [72, 73]). Because a UD represents the full probability distribution generated by an infinite number of simulation replicates, each yielding a unique movement trajectory, a sufficiently large number of replicates must be run to gain a clear, representative picture of how individual space-use patterns emerge over time, a process that is can be computationally prohibitive. In practice, transient dynamics data are frequently discarded from the main analysis when exploring UDs (but see [38]).

Some recent agent-based models have directly addressed transient space-use patterns. For example, by characterizing movement as random choices amongst nearest-neighbor lattice sites, excluding those that contain repellent environmental cues, i.e., conspecific scent marks, Potts et al. [50] demonstrated long-term instability along UD boundaries (territorial borders). This model can be subsequently reduced to a set of mean-field PDEs that analytically relate specific behavioral parameters (i.e., scent-marking rate) to summary spatial statistics (i.e., the diffusion constant of a territory border). Yet, the mathematics are incompletely understood and the analysis is technically demanding, making these models unwieldy for data fitting and hypothesis testing (but see [22] for recent advances).

In contrast to agent-based models, classical space-use models explicitly describe how a UD will evolve according to specific movement mechanisms, including group attraction and repulsion, topographical resistance, and habitat selection [3]. There, its time derivative is expressed as a Fokker–Planck equation, a modeling approach that has appeared in both discrete and continuous forms in the seminal works by Okubo [74] and Moorcroft and Lewis [1]. However, solving the Fokker–Planck equation over time (i.e., obtaining the transient UDs) is often nontrivial, whether approached analytically or numerically. As a result, modelers have conventionally adopted the simplifying steady-state assumption by setting the time derivative to zero before solving the equation.

Analyzing the existence and stability of steady states can provide insights into transient dynamics [75, 76]. Common techniques include linear analysis, amplitude equation formalism [77], and the use of energy functional minimizers [78]. Their findings are typically validated through numerical simulations starting with a small perturbation of a steady state. Although such perturbations will eventually decay, they may initially grow rapidly and persist for extended periods, resulting in long transients. The maximum instantaneous rate at which perturbations are amplified is measured by reactivity [79]. In contrast, resilience measures how quickly a stable system returns to its original state after being perturbed [80], that is, the duration of transients. Together, reactivity and resilience describe solution behavior at the temporal extremes, i.e., as time approaches zero (present) and infinity (distant future), respectively. Neither metric captures the entire range of transient behavior between these extremes. This limitation is particularly evident in movement research, such as studies examining changes in an animal’s space-use pattern following translocation, where the dynamics of interest might not be reflected in the system’s asymptotic behavior.

Several models have addressed these shortcomings of steady-state analysis by approximating transient UDs through a concatenation of sequential steady states, each computed numerically under a unique set of parameters that represents the environmental condition at a specific time [4, 7, 47]. More recently, these analyses have been enhanced through the use of numerical continuation methods [81]. However, these models implicitly assume that UDs stabilize more quickly than any fundamental changes in their underlying movement behaviors, implying a rate inequality that is possibly unrealistic [82]. The solutions likewise represent only the present ($t=0$) and long-term ($t=\infty$) states of the system, omitting what occurred in the interim.

## Methods

Classical space-use models require descriptions of the underlying movement behavior (e.g., step length, turning angle) and local responses to environmental conditions (e.g., preference for resource-rich habitats, avoidance of foreign scent marks). In animal home range and territory models, these mechanisms are typically described as a combination of random and directed movements in a Fokker–Planck equation:

1 $$\begin{aligned}\frac{\partial u\left(\mathbf{x},t\right)}{\partial t} = {\nabla }^{2}\left[d(\mathbf{x},t)u\left(\mathbf{x},t\right)\right]-\nabla \cdot \left[c(\mathbf{x},t)u\left(\mathbf{x},t\right)\overrightarrow{\mathbf{x}}\right] \\ \text{Transient term} \quad \text{Random movement} \quad \text{Directed movement}\end{aligned}$$

where $u\left(\mathbf{x},t\right)$ denotes the individual’s UD at time $t$, and $\overrightarrow{\mathbf{x}}$ is a unit vector indicating the direction of the mean center of attraction relative to the individual’s current position. In the simplest case where a central point attractor is the only source of movement bias, the local strengths of random and directed movements at time $t$, as represented by the diffusion term $d(\mathbf{x},t)$ and the advection term $c(\mathbf{x},t)$, may be reduced to constant coefficients $d$ and $c$. Here, a steady-state UD can be derived analytically [1, 7], assuming that the individual moves in a radially symmetric manner within a finite two-dimensional landscape $\Omega$ with zero-flux boundary condition.

Transient solutions of a Fokker–Planck equation typically need to be found using numerical techniques. FiPy (www.ctcms.nist.gov/fipy), developed for material science research by the National Institute of Standards and Technology (NIST), is a powerful PDE solver based on standard finite volume method that we can leverage to uncover transient UDs [53]. Written in Python, FiPy inherits the language’s growing usage, easily legible syntax, and extensibility across its large libraries of algorithms for scientific computing (e.g., NumPy, SciPy) and visualization (Matplotlib). It solves PDEs by reformulating the values of the dependent variable into discrete points on a meshed domain. The original PDE, thus reduced to a linear set of algebraic equations, can then be efficiently solved as a sparse matrix using an iterative scheme. The temporal solutions of the equation are contained within the cells that constitute the mesh.

### Background on numerical PDE solver

Several methods and tools for solving PDEs are available today (see Table in Supplementary Information). Many are impractical for use by movement modelers who wish to work at a high level of mathematical abstraction without being overwhelmed by details of numerical analysis. Some proprietary solvers (e.g., built-in toolboxes in MATLAB and Mathematica) are widely adopted, but they can be costly and lack versatility. There are also several R-based options, which may be more syntactically familiar to many quantitative ecologists. A popular solver is deSolve [54], which uses the method-of-lines (MOL) approach instead of finite volume or finite element methods. It works by first discretizing only the spatial components of a PDE, then approximating the equation with a system of ordinary differential equations that are solved forward in time. MOL produces fairly accurate solutions for 2-dimensional advection-diffusion problems [55], however, it is incapable of handling elliptic PDEs, i.e., when the time derivative is removed. Thus, it cannot be used to compare transient dynamics with respect to steady-state solutions unless the latter were already known in their analytical forms (but see R packages ReacTran and RoosSolver [54]).

In the Python environment, FiPy is a well-established solver that comes with easy customization, relatively short set-up time to program, open-source nature, and the capability to handle arbitrary combinations of elliptic, hyperbolic, and parabolic PDEs. Solution precision in FiPy can be further enhanced through a “sweeping” procedure. The overall runtime can also be shortened through parallel computing by exploiting third-party packages (e.g., PETSc, Trilinos). Models of three-dimensional space use in studies of avian and marine ecology, or those that require more topographically realistic spatial domain, can employ polygonal meshes constructed by Gmsh. Compared to several R-based methods, FiPy accommodates greater model complexity while requiring fewer lines of code to implement (Supplementary Information).

Transient space use can be modeled on a landscape that is either homogenous or heterogeneous, incorporating physical barriers (e.g., [56]) and resource fragmentation (e.g., [57]). In our models of transient space-use dynamics, we assumed movement is limited inside a bounded landscape $\Omega$ with reflective edges, thus ensuring that the area integrations of all UDs are maintained at unity. We defined $\Omega$ by constructing a mesh of either one- or two-dimensional space that consists of equidistant grid cells. At each time step, local UD solutions are determined at the cell centers, and the values between adjacent cells are estimated as flux across their boundaries (i.e., faces). When configuring the landscape, it is important to decide on the tradeoff between spatial and temporal resolutions. According to the Courant–Friedrichs–Lewy (CFL) criterion (see [58]), increasing the spatial resolution (i.e., number of grid cells) shortens the maximal allowed time-step size, thereby increasing the computational time. Placing the point attractor at a cell center helps to keep the solutions robust to approximation error even under relatively low spatial resolution.

We optimized the precision of our UD solutions by testing different approximation schemes that are available for discretizing the advective term in the Fokker–Planck equation. In all our analyses, we adopted the explicit Van Leer flux splitting method, which we found to outperform many alternatives. Hybrid, powerlaw, and related exponential-difference schemes, are arguably more versatile but have been criticized for giving qualitatively erroneous solutions under misalignment between the main advective direction and the mesh coordinates [59].

We demonstrated our modeling approach using both classical and new case studies.

## Results

### Case study 1: extending mechanistic home range (territory) analysis

We may obtain transient solutions of classical space-use models with well-known steady-state solutions. As an example, we revisited the scent-mediated territory formation model [1, 5], in which two individuals, U and V, deposit scent marks in mutual defense against foreign encroachment near their respective den sites at opposite ends of the landscape. Encounter with foreign scent marks causes one to a) “over-mark” at a rate proportional to the local density of foreign scent marks, and b) gravitate more toward its den site. On a one-dimensional landscape, this system can be modeled as follows:

2 $$\begin{aligned}\frac{\partial u\left(x,t\right)}{\partial t}\text{ } &= d{\nabla }^{2}u\left(x,t\right)-c\nabla \cdot \left[u\left(x,t\right)\text{tanh}\left(\alpha \left|x-{x}_{u}\right|\right){{\vec{x}}_{u}}q\left(x,t\right)\right],\\ \frac{\partial v\left(x,t\right)}{\partial t}\text{ } &= d{\nabla }^{2}v\left(x,t\right)-c\nabla \cdot \left[v\left(x,t\right)\text{tanh}\left(\alpha \left|x-{x}_{v}\right|\right){{\vec{x}}_{v}}p\left(x,t\right)\right],\\ \frac{dp\left(x,t\right)}{dt}\text{ } &= u\left(x,t\right)\left[1+mq\left(x,t\right)\right]-p\left(x,t\right),\\ \frac{dq\left(x,t\right)}{dt}\text{ } &= v\left(x,t\right)\left[1+mp\left(x,t\right)\right]-q\left(x,t\right).\end{aligned}$$

Here, $u\left(x,t\right)$ and $v\left(x,t\right)$ represent the two individuals’ UDs. $\nabla$ and ${\nabla }^{2}$ express their drift and diffusion processes. ${\vec{x}}_{u}$ and ${\vec{x}}_{v}$ are unit vectors pointing towards their exclusive den sites, ${x}_{u}$ and ${x}_{v}$. The inclusion of the hyperbolic tangent function removes the mathematical discontinuities at the point attractors, thereby reducing numerical noise; $\alpha$ controls the smoothness of step transition between opposite-signed unit vectors. $p\left(x,t\right)$ and $q\left(x,t\right)$ are nondimensionalized scent-mark densities associated with U and V, respectively; deposited marks decay at a unit rate over time. $m$ denotes the strength of the overmarking in response to foreign scent marks. Temporal variations in scent-mark densities are governed by ordinary different equations.

We initialized both individuals using the same UD centered at the origin (mimicking wildlife translocation) and ran the model forward in time. Note that although our model parameters are dimensionless, in practice, they can be specified in compatible units. For example, $c$ can be measured in km per day, $d$ in km^2^ per day, and the domain size in km^2^. The sequential UDs then represent space-use variation across fractional days. If we assumed these units in our model, under the constraint of the CFL criterion, each time step would last around 0.0014 day (2 min) and the terminal time (time step 10,000) would correspond to roughly 14 days. Finally, we examined the system’s convergence toward previously published steady-state solutions.

**Fig. 1:**
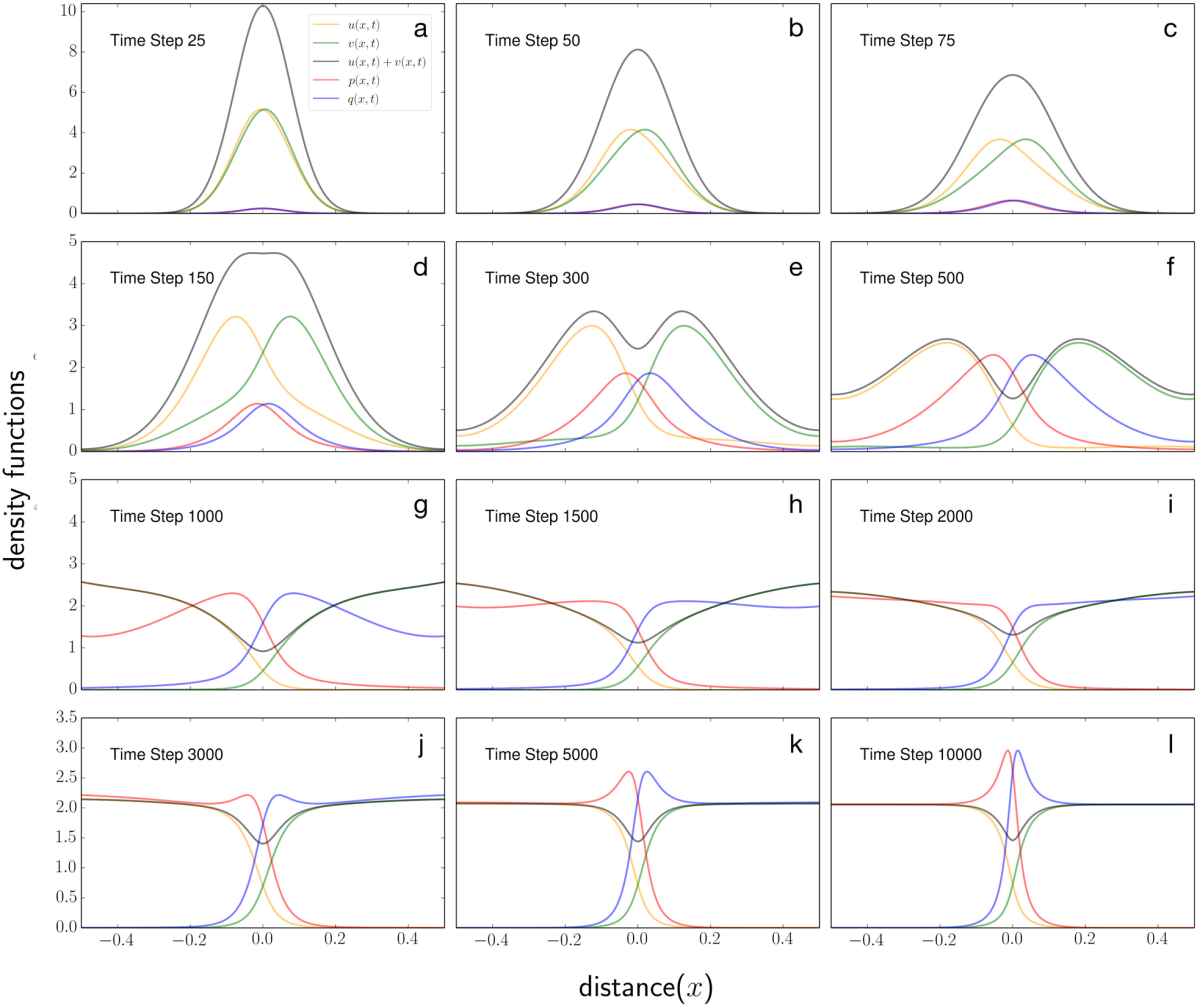
Territorial separation of two individuals (or individual packs) on a one-dimensional landscape in avoidance of scent marks deposited by one another in the environment. Den sites are located at the two ends of the landscape. The yellow and green lines represent the individual UDs; their sum is shown in black. The respective individual scent-mark densities are displayed by the red and blue lines. The process of spatial segregation during the initial 10,000 time steps post-release is captured by the transient UD dynamics; mutually deterrent boundary markings continue to accumulate long after space-use convergence

Our numerical solutions showed that the two scent-avoidant individuals released from the same site will eventually reach the steady-state UDs (Fig. 1). The space-use patterns recreated the classic findings in White et al. [5] and Moorcroft and Lewis [1]: there exists a mutually avoided area separating the territories (a “buffer zone”), which is maintained by collective scent marks.

The transient UDs revealed additional details (Fig. 1). They showed that the buffer zone appeared relatively early during the process of territorial separation (Fig. 1e). Surprisingly, each animal briefly increased its presence in areas close to its rival’s den site, before rapidly retreating once the owner’s markings began to accumulate (Fig. 1e–h). We further observed that, over time, the interior of each territory is used more uniformly, and the activity level inside the buffer zone may also increase (Fig. 1g-i). Finally, the build-up of scent marks inside the buffer zone continues long after both UDs have stabilized (Fig. 1j–l). This suggests that, by measuring the markings directly, e.g., using chemical assays or biomarkers, one may estimate territory persistence, which could be indicative of population viability.

**Fig. 2:**
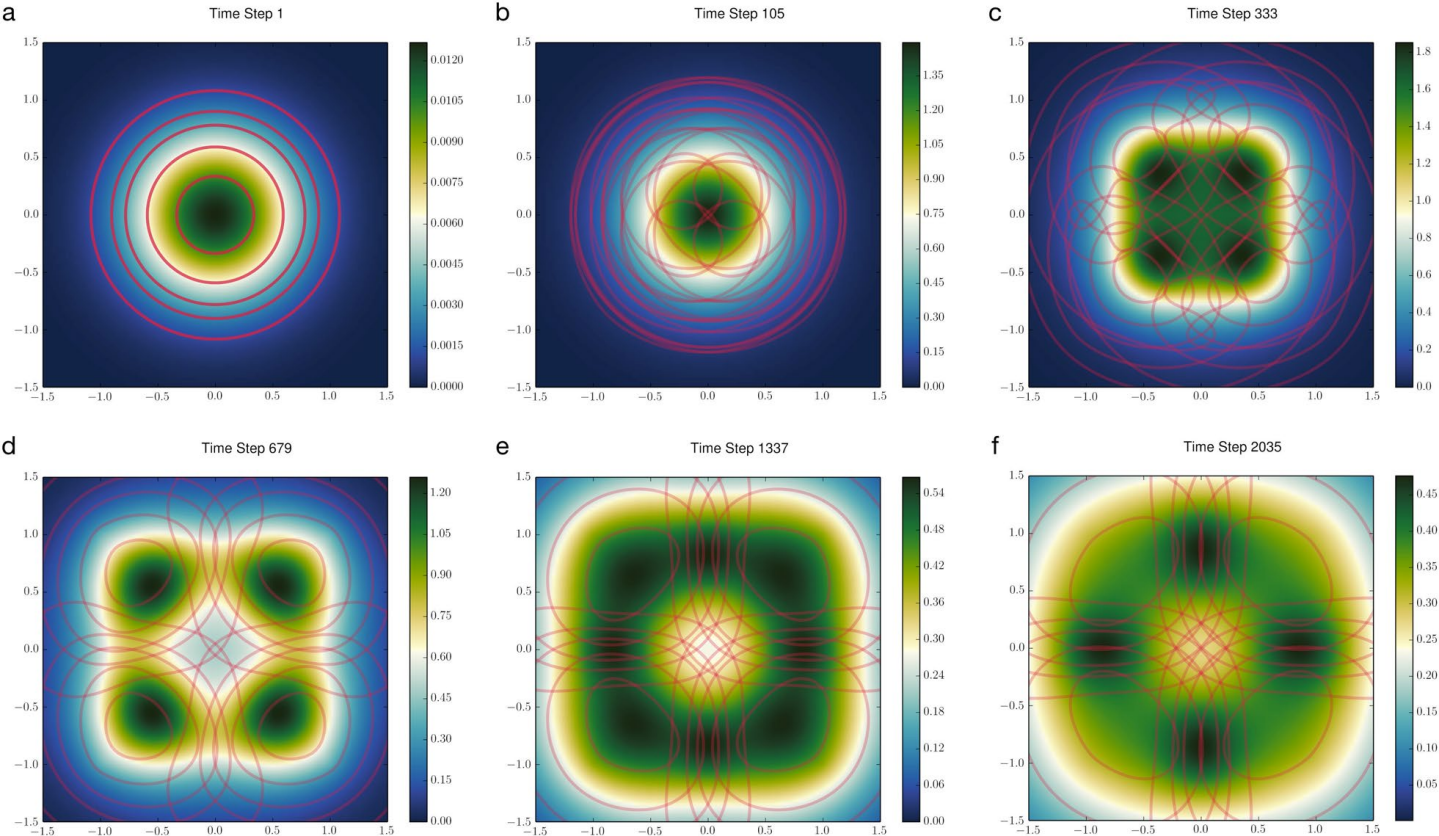
Territorial separation of four individual (or individual packs) on a two-dimensional landscape in avoidance of scent marks deposited by all others in the environment. Den sites are located at (± 1, ± 1). Red lines are contour maps illustrating each individual’s UDs at the 20%, 50%, 70%, 80%, and 90% probability levels. The color gradient shows the accumulative scent-mark densities

We can also expand the model to four territorial individuals interacting on a two-dimensional landscape, all initialized at the origin with a bivariate normal UD and moving toward separate den sites under the same set of behavioral parameters (Fig. 2). At this higher spatial dimension, with a larger population size, we observed the emergence of a ring-like scent-mark distribution, which quickly disappeared as the system approached its steady state (Fig. 2d–f). In scenarios where scent marking is accompanied by pathogen shedding (see [60]), this result suggests that transient disease hotspots may form, potentially sparking cryptic outbreaks.

### Case study 2: incorporating heterogeneous landscape structures

The role of landscape heterogeneity (e.g., terrain steepness, resource gradient) in determining space-use patterns has been repeatedly explored in mechanistic home range models [4, 47, 61]. However, these studies placed little emphasis on space-use patterns beyond the vicinity of the steady-state UD. How landscape changes might accelerate, delay, or even prevent the realization of the expected UD has typically been left unanswered.

In these examples, we tracked transient UDs on heterogenous landscapes. As in Case Study 1, transient analyses are useful for exploring space-use dynamics of translocated wildlife. We defined our model domain $\Omega$ as a physical landscape with spatially fragmented resource distribution. At the start of each model, a group of individuals, with initial locations characterized by a bivariate normal UD, began relocating to an arbitrarily located home range center ${\mathbf{x}}_{u}$, which acts as a point attractor. The individuals move at a speed $\lambda \left(\mathbf{x}\right)$ that decreases with local resource density, reflecting increased residence time in resource-rich areas. This common behavior can be described by a simple equation derived from a continuous-time, correlated random walk that combines diffusive foraging and site-fidelity, i.e., an Ornstein–Uhlenbeck process [62]:

3 $$\frac{\partial u\left(\mathbf{x},t\right)}{\partial t}\text{ } = {{d}_{0}{\lambda }^{2}(\mathbf{x})\nabla }^{2}\left[u\left(\mathbf{x},t\right)\right]-{c}_{0}\lambda \left(\mathbf{x}\right)\nabla \cdot \left[u\left(\mathbf{x},t\right)\text{tanh}\left(\alpha \left|\mathbf{x}-{\mathbf{x}}_{u}\right|\right)\overrightarrow{\mathbf{x}}\right],$$

**Fig. 3:**
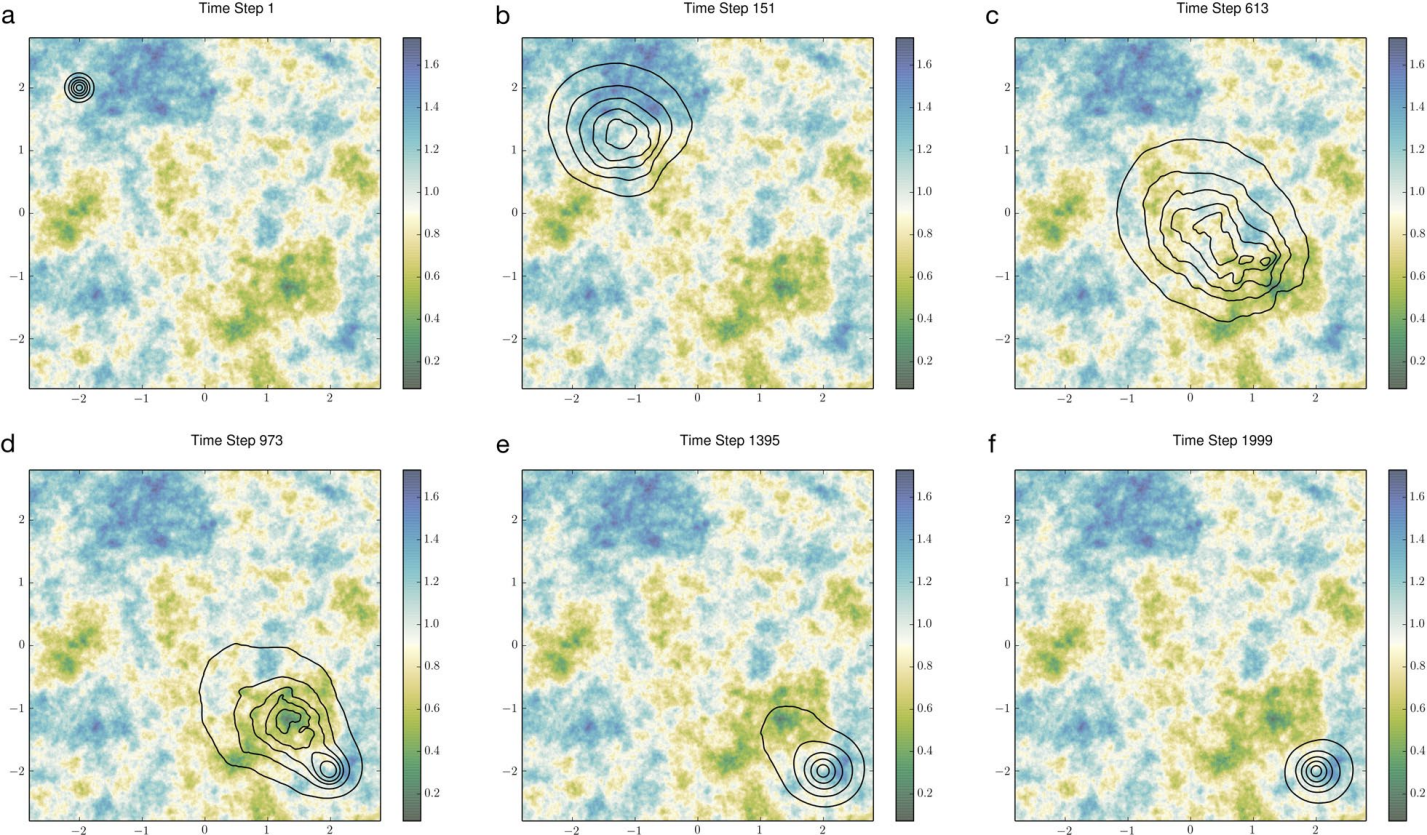
Transient space-use dynamics over the course of home range relocation on a 2D heterogeneous landscape. The movement mechanism follows a Fokker–Planck equation with spatially dependent advection and diffusion terms. A point attractor is situated in the lower-right corner (2, − 2). Black lines show contour levels of the individual’s UDs at 10%, 30%, 50%, 70%, and 90% probabilities. The color gradient illustrates the spatial distribution of local movement speed (a negative proxy for local resource availability), generated as a Gaussian field with an exponential variogram model (partial sill = 0.05, spatial correlation range = 25). The individual was assumed to slow down to forage in resource-rich patches (green) and speed up in resource-poor patches (blue)

${d}_{0}$ and ${c}_{0}$ are the baseline diffusion and advection coefficients associated with $\lambda \left(\mathbf{x}\right)=1$. Solving for the transient UDs on our realistic landscape unfolded a complex series of space-use patterns that clearly illustrated the group’s behavioral adaptation to resource availability (Fig. 3). For a brief period, two distinct UD ‘nuclei’ appeared: one at ${\mathbf{x}}_{u}$, and the other at a site some distance away (Fig. 3d).

Next, we introduced linear landscape elements to the model. In this scenario, the group faces a set of identically shaped, equally spaced movement barriers, a configuration that resembles a transecting roadway with multiple overpasses. The landscape is thus further divided into permeable and nonpermeable regions. The domain becomes $\Omega =\left\{\mathbf{x}|\mathbf{x}\in {\Omega }_{T}\right\}$, where ${\Omega }_{T}$ denotes the permeable region that is contiguously traversable. A zero-flux boundary condition was imposed on both the outer and the inner boundaries ${d\Omega }_{T}$.

**Fig. 4:**
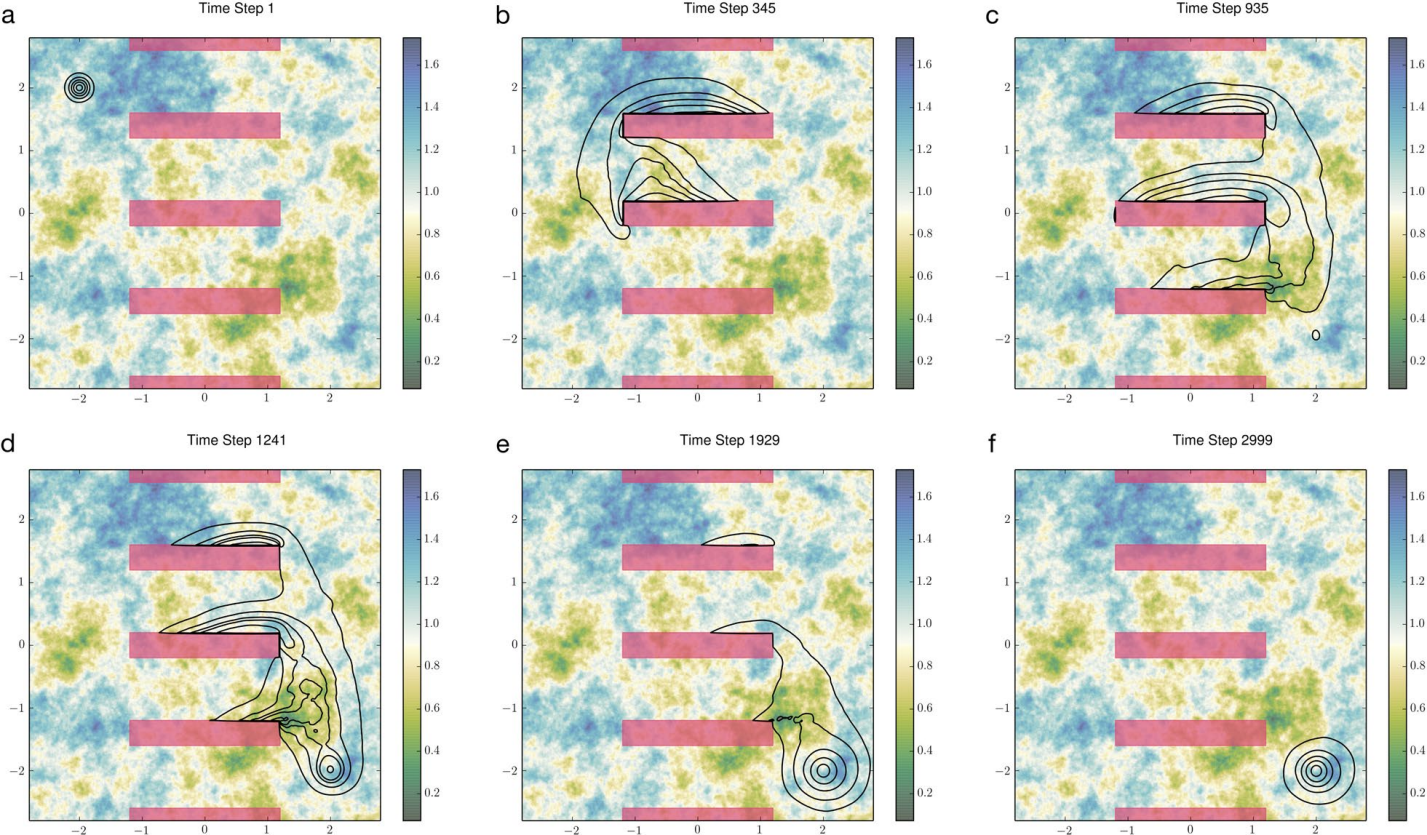
Same model as Fig. 3 except for the inclusion of linear landscape elements. Passages are partially obstructed by a set of impermeable barriers (red) that open onto multiple movement corridors of equal widths. Notably, we observed the existence of parallel realities where, for long periods of time, the individual is just as likely to be found trapped somewhat near its initial location as at its expected destination given by the steady-state UD

From the results (Fig. 4), we observed the potential of movement barriers in creating situations where, at times (Fig. 4d), the spread of likely locations (90% probability contour) covers nearly a fourth of the landscape. Since an animal's travel time often correlates with its exposure to predation and other hazards [63], our predictions of transient UDs can help inform management decisions on where and how to build movement corridors to maximize the survivorship of the target individuals.

### Case study 3: integrating space-use and population dynamics

PDEs are most appropriate for modeling continuous-time space-use patterns of individuals in an environment where the locations of movement attractors, deterrents, corridors, and barriers are either constant or vary over time in a deterministic manner. However, at a population scale, discrete stochastic events often occur due to demographic processes (e.g., births, deaths) and disease outbreaks (e.g., infection, recovery), which can lead to changes in individual movement behavior at random times and locations; moreover, future population-scale events might, in turn, be altered by these very changes [39, 64]. To capture such feedback, we introduced a hybrid method that iteratively models transient UDs in response to the simulated outcomes of a spatial agent-based model (ABM), while simultaneously simulating the ABM based on system conditions established by the transient UDs.

We demonstrated this approach by considering a house-to-house vaccination campaign at the onset of a novel outbreak. The model assumes that the vaccine is being distributed across a two-dimensional landscape via diffusive movement of a single unit of healthcare personnel from a central point of deployment ${\mathbf{x}}_{u}$. To enhance vaccination coverage, personnel movement was guided by two simple rules: 1) slow down in areas with many susceptible individuals to vaccinate, and 2) withdraw from areas with high disease prevalence, i.e., where infected individuals are numerous. The personnel’s UD is governed by the following equation:

4 $$\frac{\partial u\left(\mathbf{x},t\right)}{\partial t}\text{ } = {d}_{0}{\text{e}}^{-S(\mathbf{x},t)}{\nabla }^{2}u\left(\mathbf{x},t\right)-{c}_{0}I(\mathbf{x},\text{t})\nabla \cdot \left[u\left(\mathbf{x},t\right)\text{tanh}\left(\alpha \left|\mathbf{x}-{\mathbf{x}}_{u}\right|\right)\overrightarrow{\mathbf{x}}\right].$$

${d}_{0}$ and ${c}_{0}$ are baseline rates of diffusion and advection. $S\left(\mathbf{x},t\right)$ and $I\left(\mathbf{x},t\right)$ represent the local densities of susceptible and infected individuals at time $t$, respectively. These densities are obtained by convolving the spatial distribution of agents for each disease status with a bivariate normal contact kernel.

As $u(\mathbf{x},t)$ evolves over time, transmissions occur stochastically between infected and susceptible individuals at times $t=t'=\left(i-1\right)\tau$, where $i=1, 2,\dots , n$ and $\tau$ is the interval between consecutive ABM time steps. The probability that a susceptible individual becomes infected is given by:

5 $$\varphi \left( {\mathbf{x},t' } \right) = 1 - \exp \left[ { - \delta I\left( {\mathbf{x},t' } \right)} \right],$$

where $\delta$ denotes the rate of transmission per infectious contact. If the individual remains uninfected, they are subsequently vaccinated with probability $\omega \left(\mathbf{x},t'\right)$, which increases with both vaccine efficacy $\varepsilon$ and $u(\mathbf{x},t')$, used here as a proxy for vaccine access:

6 $$\omega \left(\mathbf{x},{t^\prime}\right)=1-\text{exp}[-\varepsilon u(\mathbf{x},{t^\prime})].$$

For computational simplicity, we assumed that the expected local wait time for vaccination is independent of the local density of susceptible individuals.

**Fig. 5:**
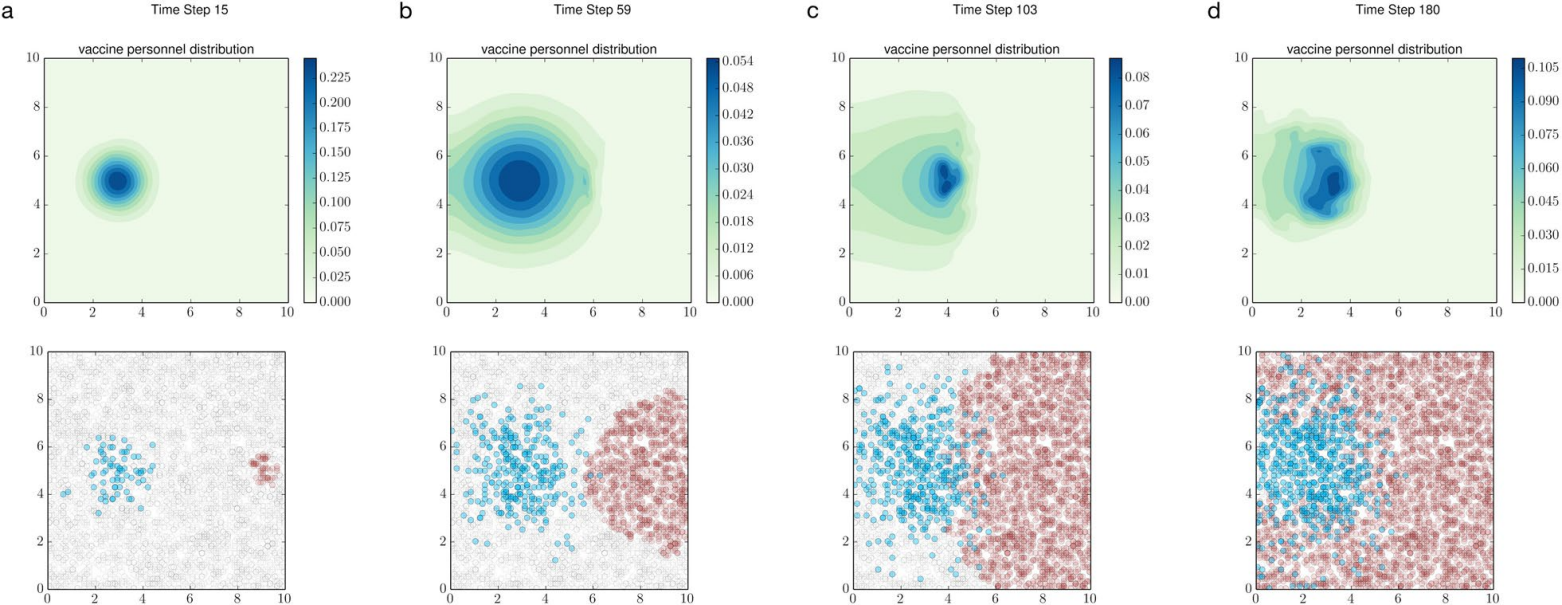
Behaviorally adaptive vaccine deployment characterized by expending more control effort in high-needs neighborhoods and withdrawing from already infected regions. Top row: The blue surfaces depict the transient UDs of the vaccination personnel in response to local disease prevalence. Bottom row: Susceptible, vaccinated, and infected individuals are shown in white, blue, and red, respectively

We simulated the discrete events described by Eqs. (5) and (6) while simultaneously solving Eq. (4) over smaller-sized time steps. The ABM outputs and the transient solutions $u\left(\mathbf{x},t\right)$ are thus tightly coupled. Our results demonstrate that a deployment strategy outlined by the above two rules can create a protective “bubble” where vaccination efforts are concentrated and disease transmissions are inhibited (Fig. 5). Combining a Fokker–Planck equation with an ABM in this manner allows us to forecast outcomes for various management strategies across diverse epidemiological contexts [32]. Crucially, this hybrid approach enables the evaluation of behaviorally complex management theories. For example, it can be used to assess whether forming a defensive perimeter with vaccinators is more effective at containing an outbreak than preemptively dispatching healthcare teams to already affected regions to halt epidemic progression.

## Discussion

In this paper, we developed a novel modeling approach that leverages existing computational tools to unmask the elusive transient solutions of classical space-use models. Our approach is intuitive, self-contained, and does not require high-level programming proficiency. We demonstrated its ability to not only advance theory in movement ecology but also to address emerging ecological and epidemiological questions.

Transient space-use dynamics merit closer attention in the movement modeling literature. In systems where the space-use patterns of one or multiple individuals are in spatiotemporal flux, transient UDs may yield greater ecological insights than traditional UD concepts rooted in steady-state assumptions. Even from a purely graphical standpoint, they impart far more information, exposing complex spatial patterns and a more elaborate sequence of events. We posit that this new dynamical perspective will have far-reaching implications across a wide range of disciplines concerned with organismal movement.

For instance, incorporating transient animal space-use dynamics can significantly enhance models of disease spread in wildlife populations. New methods that infer dynamic contact networks from animal movement trajectories, such as MoveSTIR and related frameworks [27, 65], have already laid the groundwork for mapping transient UDs to transmission opportunities (see Vargas-Soto [29]). Building on these works could lead to identification of hotspots, hot hosts, and hot time windows that disproportionately influence disease outbreaks [27]. Moreover, because transient UDs may be functions of environmental variables, these models could eventually reveal where large-scale outbreaks originate or how they are driven by host habitat conditions.

Transient space-use analysis also applies to the field of wildlife management. Consider the translocating of bears that are prone to human conflicts (e.g., [66]). The strategy's effectiveness has been debated as bears often resettle poorly, experiencing high mortality rates or attempting to return to their original place of capture. By predicting transient UDs, managers can better anticipate the frequency of hazard (e.g., road) encounters and estimate the likelihood of a successful translocation.

In this paper, we have limited our case studies to centralized movement governed by a single fixed-point attractor (e.g., a den site or a base of deployment). In natural systems, however, many point attractors can coexist, and individuals may spend considerable time traveling among them. Our approach can be generalized to model such scenarios (e.g., multi-nucleic home ranges), where the expected space-use pattern could represent a mixture of multiple transient UDs driven by different directional preferences. In addition, sensory noise and ecological perturbations, two outcomes of global environmental changes, can keep an individual’s UDs in a perpetual state of transience. The consequences of this type of spatial instability on biodiversity and public health should be further investigated. Finally, we can apply our approach to other classes of PDEs: for instance, in reaction-advection-diffusion equations, it could yield more accurate predictions of the ecological dynamics of invasive pest animals.

Transient space-use analysis typically requires a well-defined set of mechanistic assumptions. However, the latter can be difficult to ascertain. Thus, a key challenge, and an incipient area of research, is to fit mechanistic models to the copious empirical movement data currently available. Recently, Potts and Schlägel [22] developed a method to parameterize classical space-use models using step-selection analyses of animal tracking data. The fitted parameters capture an animal’s tendency to move toward or away from specific spatial features, reflecting actual movement mechanisms. In principle, one can then solve the resultant models with our approach to obtain the transient UDs, thereby integrating empirical data with real-time predictions. At the moment, this method is constrained by its assumption that each movement step represents an immediate response to local cues (e.g., conspecific markings), instead of being part of a continuous trajectory shaped by long-term objectives (e.g., home range relocation). As such, it is incompatible with space-use models that include distant attractions or repulsions. To take full advantage of transient space-use analysis, a continuous-time, multi-modal extension of step-selection analysis that accommodates remotely guided movements, as proposed by Wang et al. [67], may be necessary.

Space-use models are also fitted to movement data in hierarchical Bayesian models used to study population and disease spread, [68–70]. There, forecasts are typically generated by statistically estimating the parameters of a PDE and then solving the equation over time through manual numerical integration (e.g., [19]). Perhaps due to the difficulties involved in this process, the movement mechanisms are often highly simplified (e.g., limited to random movement). When models incorporate fine-scale environmental heterogeneity, more complex techniques, such as homogenization [18], are required, which can further deter non-expert users. Our approach to solving PDEs can enhance this hierarchical Bayesian framework in terms of both accessibility and inferential power. For instance, nesting a FiPy script that solves for transient UDs inside its Markov chain Monte Carlo (MCMC) algorithm could eliminate much of the technical burden associated with numerical analysis, enabling predictions of ecological spread under complex and realistic movement mechanisms.

## Conclusions

Transient space-use analysis can lead to reevaluations of foundational ecological concepts such as home range and territory, prompting a shift in definitions from static to dynamic constructs that more accurately reflect biological realities. It can also facilitate the integration of dynamical models developed across different spatiotemporal scales and open many new avenues of research, from exploring the links between demographic shifts and territorial spacing to examining how illness-induced behavioral changes modulate disease dynamics. By recognizing the interdependence between individual space-use dynamics and broader system-level processes, we can markedly improve our predictions of complex biological systems and formally establish movement ecology as a critical element of future ecological and epidemiology theories.

## Supplementary Information

Additional file 1.

## Data Availability

Core code for generating the figures is available at https://doi.org/10.5281/zenodo.10871588.

## References

[1] Moorcroft PR, Lewis MA. Mechanistic home range analysis (MPB-43). New Jersey: Princeton University Press; 2013.

[2] Smouse PE, et al. Stochastic modelling of animal movement. Philos Trans R Soc Lond B Biol Sci. 2010;365:2201–11. 20566497 10.1098/rstb.2010.0078 PMC2894957

[3] Potts JR, Lewis MA. How do animal territories form and change? Lessons from 20 years of mechanistic modelling. Proc Biol Sci. 2014;281:20140231. 24741017 10.1098/rspb.2014.0231 PMC4043092

[4] Bateman AW, Lewis MA, Gall G, Manser MB, Clutton-Brock TH. Territoriality and home-range dynamics in meerkats, Suricata suricatta: a mechanistic modelling approach. J Anim Ecol. 2015;84:260–71. 24995457 10.1111/1365-2656.12267

[5] White KAJ, Lewis MA, Murray JD. A model for wolf-pack territory formation and maintenance. J Theor Biol. 1996;178:29–43.

[6] Lewis MA, Murray JD. Modelling territoriality and wolf–deer interactions. Nature. 1993;366:738–40.

[7] Tao Y, Börger L, Hastings A. Dynamic range size analysis of territorial animals: an optimality approach. Am Nat. 2016;188:460–74. 27622879 10.1086/688257

[8] Nathan R, et al. Big-data approaches lead to an increased understanding of the ecology of animal movement. Science. 2022;375:eabg1780. 35175823 10.1126/science.abg1780

[9] Schick RS, et al. Understanding movement data and movement processes: current and emerging directions. Ecol Lett. 2008;11:1338–50. 19046362 10.1111/j.1461-0248.2008.01249.x

[10] Williams HJ, et al. Optimizing the use of biologgers for movement ecology research. J Anim Ecol. 2020;89:186–206. 31424571 10.1111/1365-2656.13094 PMC7041970

[11] Wilson RP, et al. Wild state secrets: ultra-sensitive measurement of micro-movement can reveal internal processes in animals. Front Ecol Environ. 2014;12:582–7.

[12] Hooten MB, Lu X, Garlick MJ, Powell JA. Animal movement models with mechanistic selection functions. Sp Stat. 2020;37: 100406.

[13] Jonsen ID, Flemming JM, Myers RA. Robust state–space modeling of animal movement data. Ecology. 2005;86:2874–80.

[14] Patterson TA, Thomas L, Wilcox C, Ovaskainen O, Matthiopoulos J. State–space models of individual animal movement. Trends Ecol Evol. 2008;23:87–94. 18191283 10.1016/j.tree.2007.10.009

[15] Boyce MS, McDonald LL. Relating populations to habitats using resource selection functions. Trends Ecol Evol. 1999;14:268–72. 10370262 10.1016/s0169-5347(99)01593-1

[16] Avgar T, Potts JR, Lewis MA, Boyce MS. Integrated step selection analysis: bridging the gap between resource selection and animal movement. Methods Ecol Evol. 2016;7:619–30.

[17] Hooten MB, Johnson DS, McClintock B, Morales J. Animal movement: Statistical models for telemetry data. (2017). 10.1201/9781315117744.

[18] Hooten MB, Garlick MJ, Powell JA. Computationally efficient statistical differential equation modeling using homogenization. J Agric Biol Environ Stat. 2013;18:405–28.

[19] Hefley TJ, Hooten MB, Russell RE, Walsh DP, Powell JA. When mechanism matters: Bayesian forecasting using models of ecological diffusion. Ecol Lett. 2017;20:640–50. 28371055 10.1111/ele.12763

[20] Garlick MJ, Powell JA, Hooten MB, MacFarlane LR. Homogenization, sex, and differential motility predict spread of chronic wasting disease in mule deer in southern Utah. J Math Biol. 2014;69:369–99. 23846241 10.1007/s00285-013-0709-z

[21] Potts JR, Börger L. How to scale up from animal movement decisions to spatiotemporal patterns: an approach via step selection. J Anim Ecol. 2023;92:16–29. 36321473 10.1111/1365-2656.13832 PMC10099581

[22] Potts JR, Schlägel UE. Parametrizing diffusion-taxis equations from animal movement trajectories using step selection analysis. Methods Ecol Evol. 2020;11:1092–105.

[23] Fieberg J, Kochanny CO. Quantifying home-range overlap: the importance of the utilization distribution. J Wildl Manage. 2005;69:1346–59.

[24] Benhamou S, Riotte-Lambert L. Beyond the utilization distribution: identifying home range areas that are intensively exploited or repeatedly visited. Ecol Modell. 2012;227:112–6.

[25] Hoyt JR, et al. Cryptic connections illuminate pathogen transmission within community networks. Nature. 2018;563:710–3. 30455422 10.1038/s41586-018-0720-z

[26] Epstein JH, et al. Nipah virus dynamics in bats and implications for spillover to humans. Proc Natl Acad Sci U S A. 2020;117:29190–201. 33139552 10.1073/pnas.2000429117 PMC7682340

[27] Wilber MQ, et al. A model for leveraging animal movement to understand spatio-temporal disease dynamics. Ecol Lett. 2022;25:1290–304. 35257466 10.1111/ele.13986

[28] Teitelbaum CS, et al. North American wintering mallards infected with highly pathogenic avian influenza show few signs of altered local or migratory movements. Sci Rep. 2023;13:14473. 37660131 10.1038/s41598-023-40921-z PMC10475108

[29] Vargas Soto JS, et al. Correlated host movements can reshape spatio-temporal disease dynamics: modeling the contributions of space use to transmission risk using movement data. bioRxiv 2024.04.16.589740 (2024) 10.1101/2024.04.16.589740.

[30] Perkins TA, et al. Calling in sick: impacts of fever on intra-urban human mobility. Proc Biol Sci. 2016;283:1834. 10.1098/rspb.2016.0390 PMC4947886 27412286

[31] Ribeiro R, et al. Incorporating environmental heterogeneity and observation effort to predict host distribution and viral spillover from a bat reservoir. Proc Biol Sci. 2023;290:20231739. 37989240 10.1098/rspb.2023.1739 PMC10688441

[32] Tao Y, Shea K, Ferrari M. Logistical constraints lead to an intermediate optimum in outbreak response vaccination. PLoS Comput Biol. 2018;14: e1006161. 29791432 10.1371/journal.pcbi.1006161 PMC5988332

[33] Fleming CH, et al. Rigorous home range estimation with movement data: a new autocorrelated kernel density estimator. Ecology. 2015;96:1182–8. 26236833 10.1890/14-2010.1

[34] Silva I, et al. Autocorrelation-informed home range estimation: a review and practical guide. Method Ecol Evol. 2022;13:534–44.

[35] Wang Y, Ding Y, Shahrampour S. TAKDE: temporal adaptive kernel density estimator for real-time dynamic density estimation. In: IEEE Trans. Patt Ana Mach Intel (2023). 10.1109/TPAMI.2023.3297950 37478030

[36] Börger L, et al. An integrated approach to identify spatiotemporal and individual-level determinants of animal home range size. Am Nat. 2006;168:471–85. 17004219 10.1086/507883

[37] Keating KA, Cherry S. Modeling utilization distributions in space and time. Ecology. 2009;90:1971–80. 19694144 10.1890/08-1131.1

[38] Signer J, et al. Simulating animal space use from fitted integrated step-selection functions (iSSF). Method Ecol Evol. 2024;15:43–50.

[39] Tao Y, Hite JL, Lafferty KD, Earn DJD, Bharti N. Transient disease dynamics across ecological scales. Theor Ecol. 2021. 10.1007/s12080-021-00514-w. 34075317 10.1007/s12080-021-00514-w PMC8156581

[40] Russell JC, Morland AJC, MacKay JWB. Random exploratory behaviour by individual island colonists. New Zeal J Ecol. 2011;35:193.

[41] Hastings A, et al. Transient phenomena in ecology. Science. 2018;361:eaat6412. 30190378 10.1126/science.aat6412

[42] Morozov A, et al. Long transients in ecology: theory and applications. Phys Life Rev. 2020;32:1–40. 31982327 10.1016/j.plrev.2019.09.004

[43] Reimer JR, et al. Noise can create or erase long transient dynamics. Theor Ecol. 2021;14:685–95.

[44] Daversa DR, Fenton A, Dell AI, Garner TWJ, Manica A. Infections on the move: how transient phases of host movement influence disease spread. Proc Biol Sci. 2017;284:20171807. 29263283 10.1098/rspb.2017.1807 PMC5745403

[45] Giuggioli L, Potts JR, Rubenstein DI, Levin SA. Stigmergy, collective actions, and animal social spacing. Proc Natl Acad Sci U S A. 2013;110:16904–9. 24082100 10.1073/pnas.1307071110 PMC3801015

[46] Lewis MA, White KAJ, Murray JD. Analysis of a model for wolf territories. J Math Biol. 1997;35:749–74.

[47] Moorcroft PR, Lewis MA, Crabtree RL. Mechanistic home range models capture spatial patterns and dynamics of coyote territories in Yellowstone. Proc Biol Sci. 2006;273:1651–9. 16769637 10.1098/rspb.2005.3439 PMC1704082

[48] Palmer MS, et al. Dynamic landscapes of fear: understanding spatiotemporal risk. Trends Ecol Evol. 2022;37:911–25. 35817684 10.1016/j.tree.2022.06.007

[49] Zeigler SL, Fagan WF. Transient windows for connectivity in a changing world. Mov Ecol. 2014;2:1. 25520812 10.1186/2051-3933-2-1 PMC4267606

[50] Potts JR, Harris S, Giuggioli L. Quantifying behavioral changes in territorial animals caused by sudden population declines. Am Nat. 2013;182:E73-82. 23933730 10.1086/671260

[51] Potts JR, Petrovskii SV. Fortune favours the brave: Movement responses shape demographic dynamics in strongly competing populations. J Theor Biol. 2017;420:190–9. 28322873 10.1016/j.jtbi.2017.03.011

[52] Tao Y, et al. Causes of delayed outbreak responses and their impacts on epidemic spread. J R Soc Interface. 2021;18:20200933. 33653111 10.1098/rsif.2020.0933 PMC8086880

[53] Guyer JE, Wheeler D, Warren JA. FiPy: Partial Differential Equations with Python. Comput Sci Eng. 2009;11:6–15.

[54] Soetaert K, Cash J, Mazzia F. Solving ordinary differential equations in R. in solving differential equations in R. Berlin: Springer; 2012.

[55] Selçuk N, Tarhan T, Tanrıkulu S. Comparison of method of lines and finite difference solutions of 2-D Navier-Stokes equations for transient laminar pipe flow. Inter J Num Meth Engin. 2002;53:1615–28.

[56] Börger L. Stuck in motion? Reconnecting questions and tools in movement ecology. J Anim Ecol. 2016;85:5–10. 26768334 10.1111/1365-2656.12464

[57] Tao Y, et al. Landscape fragmentation overturns classical metapopulation thinking. Proc Natl Acad Sci U S A. 2024;121: e2303846121. 38709920 10.1073/pnas.2303846121 PMC11098110

[58] Bennett DA, Wenwu T. Modelling adaptive, spatially aware, and mobile agents: Elk migration in Yellowstone. Inter J Geogr Info Sci. 2006;20:1039–66.

[59] Leonard BP, Drummond JE. Why you should not use ‘hybrid’, ‘power-law’ or related exponential schemes for convective modelling—there are much better alternatives. Int J Numer Method Fluids. 1995;20:421–42.

[60] Huang MHNJ, et al. Expanding CWD disease surveillance options using environmental contamination at deer signposts. Ecol Solu Evid. 2024;5: e12298.

[61] Wang M, Grimm V. Home range dynamics and population regulation: An individual-based model of the common shrew Sorex araneus. Ecol Modell. 2007;205:397–409.

[62] Blackwell PG. Random diffusion models for animal movement. Ecol Model. 1997;100:87–102.

[63] Latham ADM, Latham MC, Boyce MS, Boutin S. Movement responses by wolves to industrial linear features and their effect on woodland caribou in northeastern Alberta. Ecol Appl. 2011;21:2854–65.

[64] Morales JM, et al. Building the bridge between animal movement and population dynamics. Phil Trans Roy Soc B. 2010;365:2289–301. 20566505 10.1098/rstb.2010.0082 PMC2894961

[65] Yang A, et al. Deriving spatially explicit direct and indirect interaction networks from animal movement data. Ecol Evol. 2023;13: e9774. 36993145 10.1002/ece3.9774 PMC10040956

[66] Alldredge MW, et al. Evaluation of translocation of black bears involved in human–bear conflicts in South-central Colorado. Wild Soc Bull. 2015;39:334–40.

[67] Wang YS, Blackwell PG, Merkle JA, Potts JR. Continuous time resource selection analysis for moving animals. Methods Ecol Evol. 2019;10:1664–78.

[68] Wikle C, Hooten M. Hierarchical Bayesian spatio-temporal models for population spread. (2006).

[69] Zou J, Karr AF, Datta G, Lynch J, Grannis S. A Bayesian spatio–temporal approach for real–time detection of disease outbreaks: a case study. BMC Med Inform Decis Mak. 2014;14:108. 25476843 10.1186/s12911-014-0108-4 PMC4267748

[70] Wikle C. Hierarchical Bayesian models for predicting the spread of ecological processes. Ecology. 2003;84:1382–94.

[71] Potts JR, Giunta V, Lewis MA. Beyond resource selection: emergent spatio–temporal distributions from animal movements and stigmergent interactions. Oikos. 2022;2022: e09188.

[72] Van Moorter B, et al. Memory keeps you at home: a mechanistic model for home range emergence. Oikos. 2009;118:641–52.

[73] Riotte-Lambert L, Benhamou S, Chamaillé-Jammes S. How memory-based movement leads to nonterritorial spatial segregation. Am Nat. 2015;185:E103–16. 25811090 10.1086/680009

[74] Okubo A. Diffusion and ecological problems: mathematical models. Verlag: Springer; 1980.

[75] Stott I, Townley S, Hodgson DJ. A framework for studying transient dynamics of population projection matrix models. Ecol Lett. 2011;14:959–70. 21790932 10.1111/j.1461-0248.2011.01659.x

[76] Capdevila P, Stott I, Beger M, Salguero-Gómez R. Towards a comparative framework of demographic resilience. Trends Ecol Evol. 2020;35:776–86. 32482368 10.1016/j.tree.2020.05.001

[77] Cross MC, Hohenberg PC. Pattern formation outside of equilibrium. Rev Mod Phys. 1993;65:851–1112.

[78] Giunta V, Hillen T, Lewis M, Potts JR. Local and global existence for nonlocal multispecies advection-diffusion models. SIAM J Appl Dyn Syst. 2022;21:1686–708.

[79] Neubert MG, Caswell H. Alternatives to resilience for measuring the responses of ecological systems to perturbations. Ecology. 1997;78:653–65.

[80] Holling CS. Resilience and stability of ecological systems. (1973).

[81] Breden M, Kuehn C, Soresina C. On the influence of cross-diffusion in pattern formation. J Comput Dyn. 2021;8:213.

[82] Benhamou S. Of scales and stationarity in animal movements. Ecol Lett. 2014;17:261–72. 24350897 10.1111/ele.12225

