# Supplementary material for: Towards transient space-use dynamics: re-envisioning models of utilization distribution and their applications
Source: Mov Ecol. 2025 Feb 28;13:12. doi: 10.1186/s40462-025-00538-5 (PMC11869446; doi:10.1186/s40462-025-00538-5)
Supplement: Supplementary file 1 — Additional file 1. [file 40462_2025_538_MOESM1_ESM.docx]

Supplementary Information

| Function/  Package/  Toolbox | Language | Method | Open  Source | Spatial  Dimension | Spatial  Heterogeneity | UD + ABM | Coding  Level Required:  1, 2, 3, 4  (1=low  2=medium/low  3=medium  4=high) |
| --- | --- | --- | --- | --- | --- | --- | --- |
| Pdepe | Matlab | Finite Difference | No | 1D | No | No | 1 |
| PDE Toolbox | Matlab | Finite  Element | No | 2D, 3D | Yes | No | 3 |
| NDSolve | Wolfram Mathematica | Finite Elements/  Finite  Difference | No | 1D, 2D | No | Yes | 1 |
| ReacTran | R | Finite Difference/Finite Volume | Yes | 1D, 2D, 3D | No | No | 3 |
| py-pde | Python | Finite Difference | Yes | 1D, 2D | No | No | 3 |
| PolyFEM | C++/  Python | Finite Elements | Yes | 1D, 2D, 3D | No | No | 1 |
| DifferentialEquations.jl | Julia | Multiple | Yes | 1D, 2D, 3D | Yes | Yes | 4 |
| FiPy | Python | Finite Volume | Yes | 1D, 2D, 3D | Yes | Yes | 2 |
| Dedalus | Python | Spectral | Yes | nD | No | Yes | 4 |

Table. Summary of actively maintained numerical tools accessible to general movement modelers for solving transient solutions in systems of partial differential equations (PDEs). All the listed numerical tools require only an introductory knowledge of numerical methods and can be used to solve Equation (1).
